# Whole-genome analysis of coxsackievirus B3 reflects its genetic diversity in China and worldwide

**DOI:** 10.1186/s12985-022-01796-0

**Published:** 2022-04-18

**Authors:** Qian Yang, Dongmei Yan, Yang Song, Shuangli Zhu, Yun He, Zhenzhi Han, Dongyan Wang, Tianjiao Ji, Yong Zhang, Wenbo Xu

**Affiliations:** 1 WHO WPRO Regional Polio Reference Laboratory, National Health Commission Key Laboratory for Medical Virology, National Institute for Viral Disease Control and Prevention, Chinese Center for Disease Control and Prevention, 155 Changbai Road, Beijing, 102206 People’s Republic of China; 2 Center for Biosafety Mega-Science, Chinese Academy of Sciences, Wuhan, 430071 People’s Republic of China; 3 Shandong First Medical University & Shandong Academy of Medical Sciences, 619 Changcheng Road, Taian, 271016 People’s Republic of China

**Keywords:** Coxsackievirus B3 (CVB3), Genome, Recombination lineages, Genetic diversity

## Abstract

**Background:**

Coxsackievirus B3 (CVB3) has emerged as an active pathogen in myocarditis, aseptic meningitis, hand, foot, and mouth disease (HFMD), and pancreatitis, and is a heavy burden on public health. However, CVB3 has not been systematically analyzed with regard to whole-genome diversity and recombination. Therefore, this study was undertaken to systematically examine the genetic characteristics of CVB3 based on its whole genome.

**Methods:**

We combined CVB3 isolates from our national HFMD surveillance and global sequences retrieved from GenBank. Phylogenetic analysis was performed to examine the whole genome variety and recombination forms of CVB3 in China and worldwide.

**Results:**

Phylogenetic analysis showed that CVB3 strains isolated worldwide could be classified into clusters A–E based on the sequence of the entire *VP1* region. The predominant CVB3 strains in China belonged to cluster D, whereas cluster E CVB3 might be circulated globally compared to other clusters. The average nucleotide substitution rate in the *P1* region of CVB3 was 4.82 × 10^–3^ substitutions/site/year. Myocarditis was more common with cluster A. Clusters C and D presented more cases of acute flaccid paralysis, and cluster D may be more likely to cause HFMD. Multiple recombination events were detected among CVB3 variants, and there were twenty-three recombinant lineages of CVB3 circulating worldwide.

**Conclusions:**

Overall, this study provides full-length genomic sequences of CVB3 isolates with a wide geographic distribution over a long-term time scale in China, which will be helpful for understanding the evolution of this pathogen. Simultaneously, continuous surveillance of CVB3 is indispensable to determine its genetic diversity in China as well as worldwide.

**Supplementary Information:**

The online version contains supplementary material available at 10.1186/s12985-022-01796-0.

## Background

Enteroviruses (EVs) are important etiological agents that can cause a wide spectrum of diseases in young children, ranging from febrile illness, hand, foot, and mouth disease (HFMD), herpangina, encephalitis, acute flaccid paralysis (AFP), and even death [1–4]. EVs belong to the genus *Enterovirus* in the family *Picornaviridae* and order *Picornavirales*. Human EVs comprises four species, namely, EV-A, EV-B, EV-C, and EV-D [1–4]. To date, EV-B includes 63 serotypes, including the coxsackievirus group A (CVA, serotype 9), coxsackievirus group B (CVB, serotypes 1–6), echovirus (serotypes 1–7, 9, 11–21, 24–27, and 29–33), EV-B69, EV-B73–B75, EV-B77–B88, EV-B93, EV-B97–B98, EV-B100–B101, EV-B106–B107, and EV-B111; and non-human EVs: EV-B110, EV-B112–114 [5].

Coxsackievirus B3 (CVB3) was first reported in Connecticut, the USA in 1949, and has emerged as an active pathogen in myocarditis [6], aseptic meningitis (AM), HFMD [7, 8], and pancreatitis [9], causing a heavy burden on society. CVB3 infection is the most common cause of viral myocarditis, characterized by viral infection and myocardial inflammation [10, 11]. However, there are no vaccines or antiviral drugs available to treat CVB3 infections.

Generally, EVs were considered to have only one ORF, but a second ORF was characterized in the EV genome and its encoded protein, ORF2p [12], was found to be crucial for viral intestinal infection. In CVB3, this ORF was found to be span ATG^589^ nt to TGA^792^ nt in the prototype strain (Nancy). The first ORF encodes 11 proteins, including VP1 to VP4, 2A to 2C, and 3A to 3D. The amino acid sequence of the ORF2 product, ORF2p, is highly conserved among various EVs (EV-A, B, and C), suggesting an important role for ORF2p in EV replication or transmission.

HFMD is a viral infectious disease in young children, particularly in those younger than 5 years of age, and is associated with different types of EVs, notably enterovirus A71 (EV-A71) and Coxsackievirus A16 (CVA16) [13–15]. However, non-EV-A71 and non-CVA16 EVs became the dominant pathogens in the HFMD spectrum from 2013 in the mainland China [1, 3]. Similarly, CVB3 is becoming a vital pathogen in the HFMD spectrum [8]. The prevalence of HFMD caused by CVB3 also increases the risk of serious diseases such as myocarditis.

Frequent recombination events and mutations in EVs have been recognized as the main mechanisms for the high rate of evolution observed, which enables them to rapidly respond and adapt to new environmental challenges [16]. Recombination is a frequent inter- and intra-typic event in EVS and is an important cause for the emergence of new EV lineages and types [17].

Relying on our three-level HFMD surveillance laboratory network in China, we obtained temporal and geographical representative CVB3 sequences isolated from patients with HFMD and healthy children. To date, there has been no systematic analysis of the whole-genome variety and recombination forms of CVB3 in China and worldwide. Therefore, we performed a large-scale, (near) full-length genetic analysis of global and Chinese CVB3 isolates, including 26 newly sequenced samples collected extensively in the mainland of China between 2012 and 2020 CVB3 sequences retrieved from the GenBank database.

## Methods

### Virus isolation

Clinical specimens were collected from patients with HFMD and from healthy children in the mainland of China. The clinical specimens from patients with hand, foot and mouth disease (HFMD) were collected according to the standard protocols of the National guideline for HFMD (http://www.gov.cn/gzdt/2009-06/04/content_1332078.htm). According to the National HFMD pathogen surveillance system, which was established in 2008 in the mainland of China, HFMD cases were reported by the provincial CDC and the representative samples were sent to the national HFMD laboratory for confirmation. These representative samples sent by the provincial CDC are the source of samples for this study. Real-time reverse transcription-polymerase chain reaction (RT-PCR) was performed to screen for EV-A71, CVA16, and other EVs as previously described [10]. Non-EV-A71 and non-CVA16 EV-positive samples were selected for further analysis. All samples were processed according to the standard and previously described protocols [9]. The viruses were isolated from the original clinical specimens by propagation in human rhabdomyosarcoma (RD) and human larynx carcinoma (HEp-2) cells following conventional methods [11]. These two cell lines were obtained from the WHO Global Poliovirus Specialized Laboratory, USA, and were originally purchased from the American Type Culture Collection.

### Full-length genome sequencing of CVB3 isolates

We selected the non-EV-A71 and non-CVA16 EV-positive samples, and extracted the viral RNA using a QIAamp Viral RNA Mini Kit (Qiagen, Valencia, CA, USA). Reverse transcription polymerase chain reaction (RT-PCR) was performed to amplify the entire *VP1* capsid region using the PrimeScript One Step RT-PCR Kit Ver. 2 (TaKaRa, Dalian, China) and the primers E490/E492 were used to amplify a portion of the *VP1* region [8]. Preparation of RT-PCR reactions and amplification profiles were based on previously reported protocols [8]. PCR products were purified using a QIAquick PCR Purification Kit (Qiagen, Germany). The amplicons were bi-directionally sequenced using an ABI 3130 Genetic Analyzer (Applied Biosystems, USA). Sequences were analyzed using the EV Genotyping Tool and BLAST server [18]. To obtain the entire genome sequence of CVB3, specific primers were used based on a previous study [8]. The obtained sequences were deposited in GenBank under the accession numbers OK632332–OK632353, OK643871–OK643874, OK643877–OK643882. The CVB3 strains used in this study were isolated between 2012 and 2020. Twenty-six samples isolated between 2012 and 2020 were identified as CVB3. The geographic distribution map of CVB3 isolates from the mainland China was obtained from Highcharts (Grant number: 0321912045738052).

### Phylogenetic analysis of CVB3 sequences

Alignment of the nucleotide sequences from CVB3 strains was performed using the BioEdit sequence alignment editor software (version 5.0). Maximum likelihood (ML) trees were estimated using the best-fit Tamura-Nei model (TN93) + Gamma (G) parameter model of nucleotide substitution in MEGA software (version 5.03). Branch lengths of the dendrogram were determined based on the tree topology and the majority rule consensus of 1000 bootstrap replicates. Bootstrap values greater than 70% were considered statistically significant for grouping. The consensus principle of EV genogrouping is at least 15% of the difference in the entire *VP1* nucleotide sequences. The global evolutionary dynamics of CVB3 were inferred based on the entire *P1* capsid region. The correlation coefficient and regression value of each dataset were calculated using TempEst (v1.5.1) to estimate the correlation between sequence divergence and the date of isolation in each dataset [19]. The Markov chain Monte Carlo (MCMC) method implemented in BEAST (v1.7.5) was used to estimate the temporal phylogenies and rates of evolution [20]. All 84 *P1* region sequences were analyzed using the uncorrected relaxed molecular clock (exponential) and constant site tree prior to the GTR + G + I nucleotide substitution model. A Bayesian MCMC run comprised 3 × 10^8^ generations to ensure that each parameter could converge. The sampling frequency was set to 3 × 10^4^ generations. The output from BEAST was analyzed using TRACER (v1.7.1) (http://beast.community/tracer) (with estimated sample size (ESS) values higher than 200). A maximum clade credibility (MCC) tree was constructed using TreeAnnotator, and the burn-in option was used to remove the first 10% of the sampled trees; the resulting tree was visualized using FigTree (v1.4.4).

### Recombination analysis of CVB3 sequences

In this study, we scanned the entire genomic sequence of CVB3 and potential recombinants from GenBank. Briefly, the *P2* and *P3* coding sequences of these strains were analyzed using BLAST to compare their identity with the sequences from GenBank. Sequences with a similarity higher than 85 percent were considered potential parental sequences and were downloaded from GenBank. Similarity plots and bootscanning analyses were performed using Simplot (version 3.5.1; Stuart Ray, Johns Hopkins University, Baltimore, MD, USA). A sliding window of 200 nucleotides was used, moving in 20-nucleotide steps, and bootscanning analyses were performed using the neighbor-joining method [21].

## Results

### Global CVB3 epidemiology

With the rapid development of molecular biology technologies, a cDNA copy covering two-thirds of the CVB3 genome was cloned in Sweden in 1984 [22]. In 1985, the biologically active CVB3 virus (Nancy strain) cDNA, including the entire genome, was synthesized in Germany [23]. When the full-length genome of CVB3 was first reported in 1987, the number of CVB3 isolates significantly increased. CVB3 related outbreaks have been reported frequently, such as an outbreak in southern Louisiana, USA in 1959 (51 patients) [24], an outbreak in South Africa in 1984 with a variety of clinical manifestations [25], an outbreak of herpangina in Japan in 1987[26] (22 patients), an outbreak in Thailand in 1988 [27], an October 1992 outbreak of infant infection in a hospital in Beijing (35 patients) [28], a June 2005 outbreak of neonatal infections in Taiwan, China [29], 2008 outbreaks of aseptic meningitis in Shandong Province, China (81 CVB3 isolates) [30], and Hong Kong, China (69 CVB3 isolates) [31], an outbreak of HFMD in Hebei in 2012 (35 cases), and an outbreak of HFMD in Shandong in 2016 (42 patients) [8]. During the EV-D68 outbreak in 2014 in the United States, CVB3 was the most commonly identified type of EV infection [32].

To date, CVB3 strains are associated with various diseases, and the manifestations vary from mild respiratory [33], gastrointestinal infections [34, 35], herpangina [36], and hand, foot, and mouth disease [7, 8, 37], to more severe diseases such as heart disease (including viral myocarditis [6, 38], pericarditis [39], and acute myocardial infarction [40]), CNS involvement (meningitis [30, 36, 41–43], meningo-cerebellitis [11], encephalitis [44], and AFP [45–47]), and pancreatic interrelated disease (pancreatitis [48], diabetes [49]). More seriously, the virus can cause neonatal sepsis-like illness and sudden death in infected infants [38, 50] and adults with low immune function.

## Summary of CVB3 datasets

**Fig. 1:**
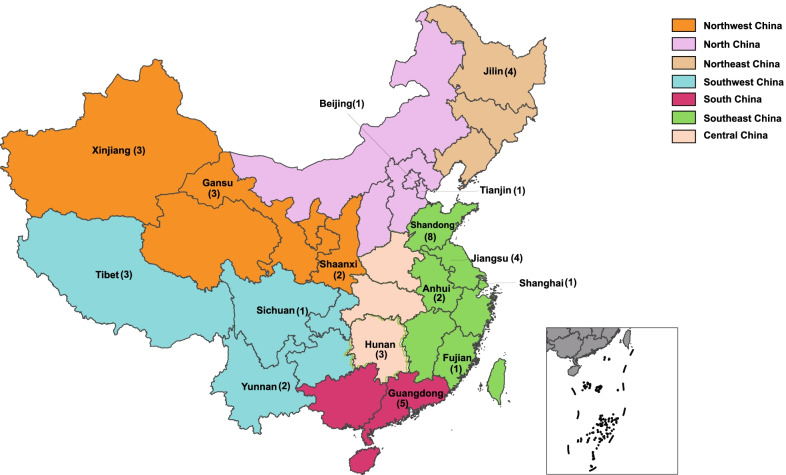
The geographical distribution whole genome of CVB3 sequences in the mainland China. Numbers in brackets represented the whole genome sequences (near the whole genome) in each provinces (municipalities, and autonomous regions) of the mainland of China

In total, 319 entire *VP1* sequences (before 2021 August 31st) were collected for the CVB3 genotyping and clinical manifestations summary. The CVB3 strains isolated between 1949 (the prototype strains) and 2020 from 17 countries, included countries in Asia ( China, Japan, India, Thailand, Uzbekistan, Indonesia), Europe (Germany, France, UK, Poland, Denmark, Russia, Romania, Moldova), North America (USA), Oceania (Australia), and Africa (Madagascar), representing wide temporal and regional distributions. In total, 84 CVB3 whole genome sequences (near the whole genome) (this study, n = 26; GenBank, n = 58) were used for the analysis. The sequence information is summarized in Additional file 1: Table S1. In total, 44 CVB3 whole genome sequences (near the whole genome) were included from the mainland of China from 2001 to 2020, in 16 provinces, municipalities, and autonomous regions, representing seven administrative regions of China: Southeast China (Shandong, Jiangsu, Shanghai, Anhui, and Fujian), Central China (Hunan), South China (Guangdong), North China (Beijing and Tianjin), Northwest China (Shaanxi, Gansu, and Xinjiang), Southwest China (Sichuan, Yunnan, and Tibet), and Northeast China (Jilin) (Fig. 1). It showed that CVB3 has been widely spread in mainland of China, covering seven geographic regions and 16 out of 31 provinces (municipalities, and autonomous regions) of the mainland of China. Most patients (19/26, 73.1%) were 1 ~ 3 years old, the median age of these patients was 2.7 years old, and all severe cases (4 cases) were under 2 years old. Men are in the majority, with a ratio of 1.6:1. Nearly 9/10 (23/26, 88.5%) cases occurred between May and August, when was spring and summer time in China. CVB3 strains isolated between 1949 (the prototype strains) and 2020 were from 12 countries and regions, including the mainland of China. A flow chart to show CVB3 sequences used in this analysis was in Additional file 2: Fig. S1.

### Five clusters were assigned based on the complete *VP1* coding region of CVB3 isolates

**Fig. 2:**
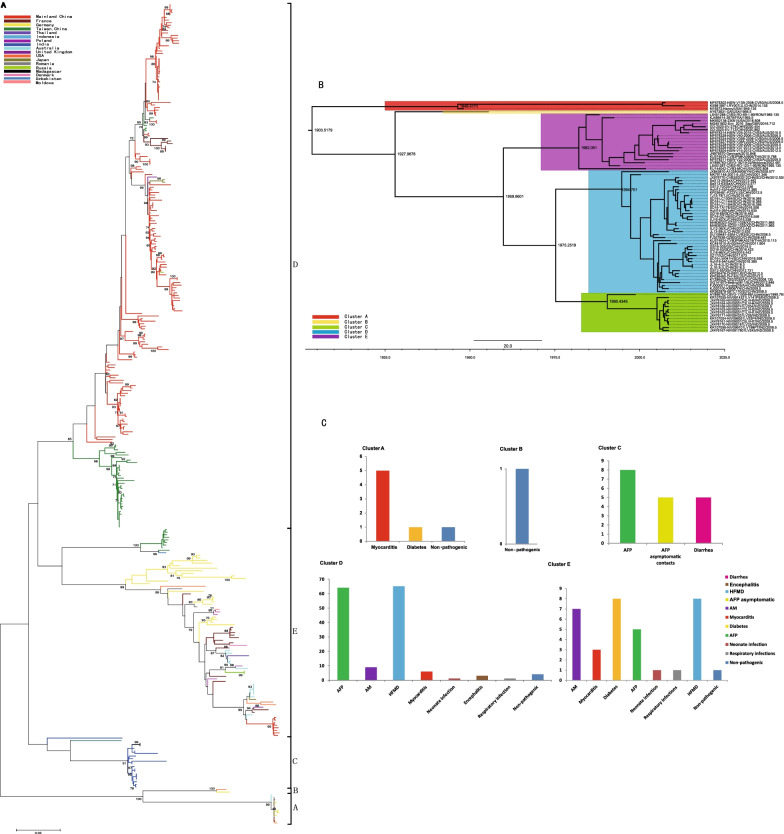
The genetic diversity and clinical manifestations distribution of CVB3 worldwide as determined in this study. **A** The maximum likelihood phylogenetic tree based on the entire *VP1* genome of 319 CVB3 genome sequences for cluster division. **B** The maximum clade credibility (MCC) phylogenetic tree based on the entire *P1* coding region of 77 CVB3 strains around the world. **C** The distribution of diseases caused by CVB3 in different clusters. The numbers of disease cases in each cluster are indicated by in the bar chart

In total, 319 entire *VP1* sequences were collected, including the mainland China (n = 167), Taiwan, China (n = 46), Japan (n = 3), India (n = 17), Thailand(n = 2), Uzbekistan (n = 1), Indonesia (n = 1), Germany (n = 27), France (n = 19), UK (n = 1), Poland (n = 4), Denmark (n = 1), Russia (n = 3), Romania (n = 2), Moldova (n = 1), USA (n = 8), Australia (n = 14), and Madagascar (n = 2). We divided the global CVB3 into five clusters (A–E) based on the entire *VP1* sequence (Fig. 2A). The cluster mean distances varied from 16.9% (clusters C to D) to 21.5% (clusters A to C). The mean genetic distance within the cluster varied from 0.6% (cluster A) to 12.0% (cluster E), indicating the reliability of genotyping. The prototype strain Nancy, which was isolated in the USA in 1949, clustered with the strains isolated in Australia, the USA, Germany, and the mainland of China to form cluster A. Cluster B included strains isolated in the USA in 1956 and in Germany in 1999. Most Indian strains, one strain from Uzbekistan, and two from Madagascar formed cluster C. Most cluster D strains were isolated in China. Cluster E comprised strains isolated from 14 countries, suggesting that cluster E might be transmitted globally. Notably, the Chinese isolates fell into clusters A, D, and E. Cluster D CVB3 was first isolated from cases of acute flaccid paralysis in 1990 and has persistently circulated in the mainland of China from 1993 to 2017, suggesting strong transmission potential. We found that the dominant cluster D had a wide temporal and geographical distribution, whereas cluster A disappeared after 2014. Emerging cluster E was only detected among HFMD cases in 2020 in one province and was possibly imported from other countries. The basic data for each cluster are summarized in Table 1. The phylogenetic analysis of CVB3 based on the ORF1sequences (near the whole genome) was also constructed, and distinct difference was not observed compared with the *VP1* tree. The five clusters could also be seen in the ORF1sequences phylogenetic trees (Additional file 3: Fig. S2).

**Table 1:** Information of 5 clusters CVB3s relying on entire *VP1* sequences

Cluster	Sequences number	Country of isolation	Year	Intra-cluster distances (%)
A	12	Australia, USA, Germany, China	1949–2014	0.6
B	2	USA, Germany	1956–1999	3.6
C	20	India, Uzbekistan, Madagascar	1999–2011	5.2
D	204	China, Thailand, Russia, Japan, France	1990–2017	8.3
E	81	China, Thailand, Indonesia, Japan, Moldova, Russia, Romania, Poland, Germany, France, Denmark, the UK, Australia, USA	1989–2020	12.0

To investigate the evolutionary history of CVB3, MCC trees (Fig. 2B) were constructed based on the entire *P1* nucleotide sequence of CVB3 strains (n = 77). The correlation coefficient was 0.74 and the R2 value was 0.54. A positive correlation was observed between genetic divergence and sampling time. The average nucleotide substitution rate for the *P1* coding region in all CVB3 strains worldwide was 4.82 × 10^–3^ substitutions/site/year (95% HPD, 3.51 × 10^–3^–6.05 × 10^–3^), which is slightly higher compared to the *P1* evolutionary rate of CV-A6 reported by others [1], indicating its rapid spreading rate. The topological structure of the MCC tree constructed using BEAST was nearly identical to that of the ML tree constructed using MEGA software. Based on our analysis, the estimated T_MRCA_ of CVB3 was in the early 1900s, corresponding to at least 40 years before the first reported detection of CVB3 in 1949. Global CVB3 strains isolated since 1903 formed two branches. Branch 1 included only cluster A, which arose in 1946, including the prototype strain Nancy. Branch 2 included cluster B, cluster C with a tMRCA that emerged in 1990, cluster D (emerged in approximately 1994), and cluster E emerging in 1982.

We also focused on the clinical manifestations of different clusters. Myocarditis (41.7%) was more common in cluster A than that in the other clusters. Cluster C (40%) and cluster D (31.4%) presented more cases of AFP compared to the other clusters. In addition, while comparing the numbers (n = 65) and proportions (31.9%) of HFMD cases among the five clusters in this study, we found that cluster D may be more likely to cause HFMD. The most common diseases in cluster E were HFMD (9.9%), diabetes (9.9%), and aseptic meningitis (8.6%) (Fig. 2C).

### Full-length genome analysis of CVB3 exhibited diverse recombination lineages in China and worldwide

**Fig. 3:**
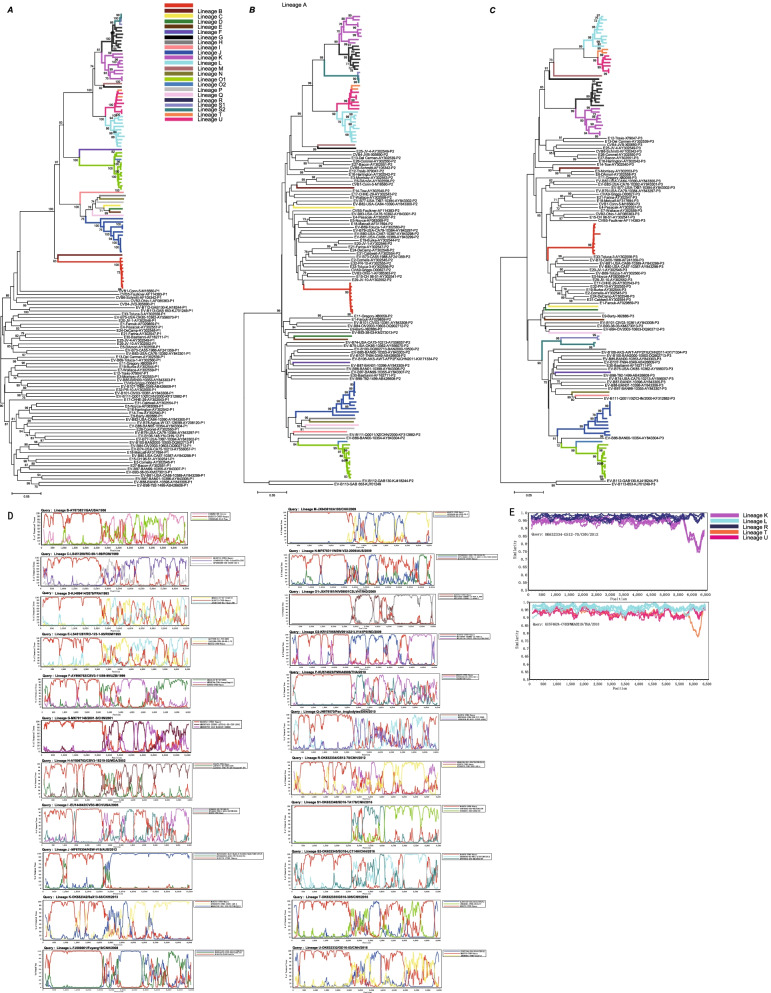
Neighbor-joining phylogenetic trees of 84 global CVB3 constructed based on the coding regions. **A** *P1* capsid region. **B** *P2* non-structural region. **C** *P3* non-structural region. The lineages are differentiated by distinct colors. **D** The diverse recombinant lineages were certified based on ORF1 sequences using bootscanning analyses. The prototype Nancy of CVB3 from lineage A was labelled red as reference. **E** Inter-lineage pairwise similarity comparison of main lineages circulating in the mainland China based on ORF1 sequences using sliding window nucleotide similarity analysis with a 200-nt window moving in 20-nt steps

Phylogenetic trees were constructed based on the entire *P1*, *P2*, and *P3* region nucleotide sequences of CVB3 strains along with the prototype of EV-B from GenBank database. Consistent with the phylogenetic tree of the *VP1* coding region, the *P1* phylogenetic tree (Fig. 3A) indicated the existence of the five CVB3 clusters in the world as expected, verifying the primary genotyping results. Unlike the *P1* phylogenetic trees, those of the *P2* and *P3* non-structural regions showed that several independent lineages clustered with the prototype strains of other EV-B prototypes rather than with the prototype of CVB3, suggesting the occurrence of recombination between CVB3 and other EV-B serotypes (Fig. 3B, C).The CVB3 whole genomes displayed significant genetic diversity in the non-structural regions, and a total of twenty-three recombinant lineages were detected (Table 2). We named the recombinant lineages based on their first isolation time. Clusters A and B had only one lineage each, consistent with the *VP1* region phylogenic tree. Cluster C contained three lineages: lineage F, lineage O1, and lineage O2. Cluster D isolates were differentiated into lineage G, lineage K, lineage L, lineage M, lineage R, lineage S1, lineage S2, lineage T, and lineage U, suggesting that at least nine different CVB3 recombinant lineages are circulating in China. Cluster E was separated into independent lineages by other EV-B prototype strains, including lineages C, D, E, H, I, J, N, P, and Q. Some recombinant lineages, such as lineages B–F, are ancient lineages from the last century and tend to be absent. The recombinant lineage J was associated with cluster E, which clearly covered several countries and was spread extensively. The diverse recombinant lineages were certified by bootscanning analyses using ORF1 sequences (Fig. 3D). The intra-lineage nucleotide similarity of lineages A, I, J, K, L, O1, O2, R, S, U, and T was between 95.8–99.9%, indicating that all the strains in the same lineage displayed high intra-lineage homology throughout the ORF1 sequences. However, when all the lineages were considered together, the diversity was obviously higher in the non-capsid regions (Fig. 3E).

**Table 2:** Information of 23 recombinant lineages of CVB3s based on *P2* and *P3* non-structural region sequences

Lineage	Cluster	Sequence number	Nucleotide mean distances (%)	Isolated countries/regions	Isolated year
A	A	10	0.46	USA, China	1949–2014
B	B	1	NA	USA	1956
C	E	1	NA	Romania	1989
D	E	1	NA	France	1993
E	E	1	NA	Romania	1995
F	C	1	NA	Uzbekistan	1999
G	D	1	NA	China	2001
H	E	1	NA	Moldova	2002
I	E	1	NA	USA	2005
J	E	11	4.14	Australia, the UK, USA, China	2006–2020
K	D	9	4.24	China	2006–2013
L	D	10	2.80	China, Thailand	2008–2012
M	D	1	NA	China	2009
N	E	1	NA	Australia	2009
O1	C	11	1.39	India	2009
O2	C	1	NA	India	2009
P	E	1	NA	Thailand	2010
Q	E	1	NA	Denmark	2010
R	D	8	2.65	China	2012–2016
S1	D	2	0.10	China	2016
S2	D	2	0.43	China	2016
T	D	2	1.84	China	2016
U	D	6	2.30	China	2016–2017

**Fig. 4:**
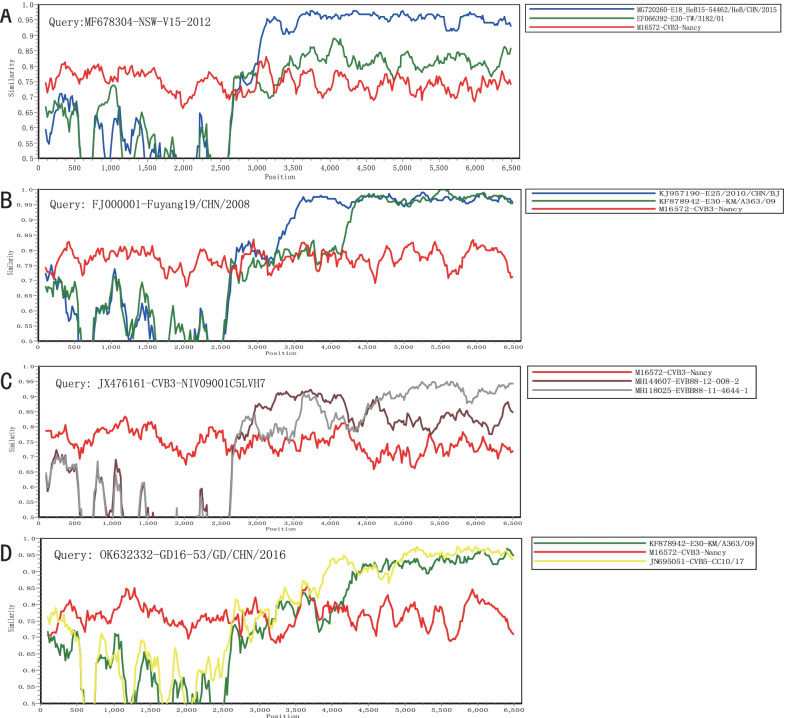
Similarity plots were constructed for detecting potential recombination detection among the screened EV-B strains and **A** lineage J, **B** lineage L, **C** lineage O1, and **D** lineage U

Previous studies have shown that EV-Bs are more susceptible to recombination compared to EV-As [51]. Regarding CVB3, many recombinant lineages, which have persisted for many years and were widespread, are still globally active. For example, lineage J included 11 strains showing high sequence identity with each other. The *P2* and *P3* non-structural coding region of lineage J, which clustered with the E18 (MG720260) and E30 (EF066392) strains, exhibit recombination activities in the evolutionary process of CVB3 (Fig. 4A). Furthermore, the non-structural genomic region of lineage L was found to have recombination breakpoints with three different EV-B serotypes: E25 (KJ957190) and E30 (KF878942) strains; in contrast, the *P1* coding region showed higher similarity with the CVB3 prototype strain (Fig. 4B). Lineage O1 showed a complex recombination with the EVB88 (MH144607, MH118025) strains in the whole *P3* region (Fig. 4C). Lineage U showed obvious recombination events, corresponding to the CVB5 (JN695051) and E30 (KF878942) strains (Fig. 4D). Therefore, the other serotypes of EV-Bs, such as echovirus (E18, E25, and E30), CVB5, and novel EV-B88 play important roles in the natural recombination of CVB3. These results indicate that inter-serotype recombination events occurred in the non-structural coding region of CVB3. The current circulating CVB3 strains are recombinants; however, the exact source of recombinant fragments was not identified; therefore, further studies are still needed to determine the EV serotype circulating simultaneously.

## Discussion

In addition to EV-A71, CVA16, CVA6, and CVA10, other EVs also account for sporadic cases of HFMD and occasional outbreak events. CVB3, which was previously detected infrequently, was recently identified as a pathogen causing HFMD outbreaks [7, 8]. Persistent outbreaks of HFMD have been causing emerging nationwide epidemics since 2007; therefore, systematic national EV surveillance is needed to comprehensively study EV pathogens. With our continuous surveillance data on CVB3-related HFMD cases collected over a 10-year period, we performed a systematic analysis of CVB3 full-length genomic characteristics and recombination forms.

EVs are characterized by their high evolutionary rates, which are largely attributed to the low fidelity of replication with error-prone RNA polymerases. The evolutionary mechanisms of EVs include mutation and recombination. Genetic mutations can change the pathogenicity, antigenicity, and host range; further, recombination occurs extremely frequently, allowing independent evolution of EV genome fragments [52].

**Fig. 5:**
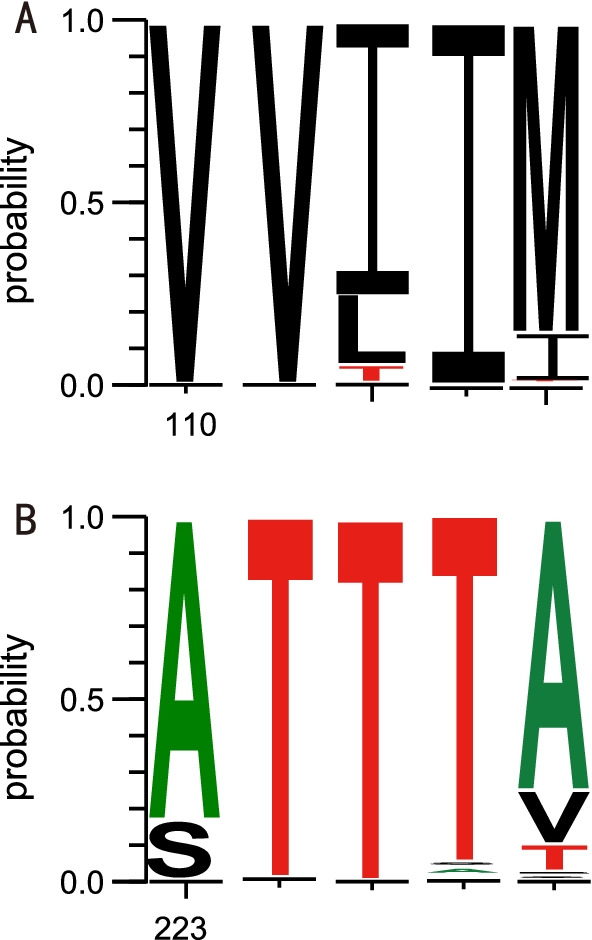
The amino acid positions of 110 (**A**) and 223 (**B**) in the VP1 region among 5 cluster CVB3 strains

CVB3 can cause various human diseases, implying multiple strains with different virulence in a single serotype. Previous studies on CVB3 with clinical descriptions indicated a potential correlation between clinical phenotypes and genetic mutations. Nucleotide substitutions in the coding region and non-coding region are reported to be related to the virulence of CVB3, and can change the degree of damage to the organs. One study identified two differences among nucleotides in untranslated regions and 8 amino acid differences in the coding regions relative to the cardiovirulent CVB3 strains. It is reported that the mutation of amino acids in P126M and D155G in the VP1 region may lead to the attenuation of CVB3 without myocardial injury [53]. Other data indicate that amino acids at positions 124–241 of VP1 in CVB3 are very important in protecting mice from death [52]. Thus, CVB3 strains can be divided into cardiovirulent strains (that are pathogenic for both the murine heart and the pancreas), pancreovirulent strains (that are pathogenic for the pancreas), and non-pathogenic strains. We compared the nucleotide and amino acid mutations across the entire VP1 region in different clusters. The results showed that the amino acid positions of 110 (χ^2^ = 449.45, *P* < 0.001) and 223 (χ^2^ = 124.54, *P* < 0.001) were significantly different among the five clusters (Fig. 5). Whether the two mutations are correlated with different types of diseases among different clusters requires further experimental research. Therefore, our further study will focus on the experimental models to precisely identify the functions of the two amino acids.

Intratypic and intertypic forms of homologous recombination are also thought to be relatively frequent evolutionary forces that affect enteroviral genomes. Although the number of whole genomes was limited, we could still find the recombination diversity in global CVB3 sequences. We allocated the worldwide CVB3 variants to twenty-three different evolutionary recombinant lineages based on non-capsid sequences. Several previous studies showed that Chinese CVB3 showed the highest similarity with the parental CVB3 in the *P1* region, with the E25 strain in the *P2-P3* region, and other strains of CVB3 showed similarity with CVB5 in the *P3* region [54, 55], which is consistent with our study. However, it remains unclear whether there are substantial differences in recombination frequencies between different EVs such as echoviruses, and whether CVB5 plays a major role in the natural recombination of CVB3.

Previous studies on EVs, such as CVA6 [1], indicated a potential correlation between clinical phenotypes and recombination. Our research does have some limitations, as there is a limited number of CVB3 sequences in each lineage, and 13 lineages have only one sequence. Therefore, we could not analyze the potential correlation between the pathogenicity and genetic recombination of CVB3.

## Conclusions

Overall, this study summarized the global CVB3 genetic diversity in both the *VP1* region and the whole genome, and the database provided insight into the global phylogenetic characteristics of CVB3. The complete genome analysis provided more comprehensive and detailed information compared to that based only on the *VP1* region. With more than half a century’s circulation, different clusters CVB3 had caused several outbreaks. Our results indicated a potential correlation between clinical phenotypes and genetic diversity. Therefore, continued surveillance is necessary to understand the complete genetic diversity of CVB3 in the mainland China as well as worldwide.

## Supplementary Information

**Additional file 1: Table S1**. List of sequences of coxsackievirus B3 used in this analysis.

**Additional file 2: Fig. S1.** A flow chart to show CVB3 sequences used in this analysis.

**Additional file 3: Fig. S2.** The maximum likelihood phylogenetic tree based on the ORF1 sequences (close to the whole genome) of 84 CVB3 genome sequences.

## Data Availability

The CVB3 nucleotide sequences determined in this study have been deposited in the GenBank nucleotide sequence database under accession numbers OK632332– OK632353, OK643871–OK643874, OK643877–OK643882.

## Abbreviations

EV: Enterovirus
HFMD: Hand, foot, and mouth disease
AFP: Acute flaccid paralysis
CVB3: Coxsackievirus B3
AM: Aseptic meningitis
RT-PCR: Real-time reverse transcription-polymerase chain reaction
RD: Rhabdomyosarcoma
HEp-2: Human larynx carcinoma
MCMC: Markov chain Monte Carlo
MCC: Maximum clade credibility
