# Supplementary material for: Whole-genome analysis of coxsackievirus B3 reflects its genetic diversity in China and worldwide
Source: Virol J. 2022 Apr 18;19:69. doi: 10.1186/s12985-022-01796-0 (PMC9014606; doi:10.1186/s12985-022-01796-0)
Supplement: Supplementary file 1 — Additional file 1: Table S1. List of sequences of coxsackievirus B3 used in this analysis. [file 12985_2022_1796_MOESM1_ESM.docx]

Table S1. The list of sequences of coxsackievirus B3 used in this analysis

| **Isolation**  **year** | **Countries or regions** | **Strain name** | **GenBank accession No.** | **Origin** | **Genotype** |
| --- | --- | --- | --- | --- | --- |
| 2014 | China | LRY007 | KX981987 | Myocarditis | A |
| 2013 | China | MKP | KJ025083 | Myocarditis | A |
| NA | Germany | 31-1-93 | AF231763 | NA | A |
| NA | Germany | P | AF231764 | NA | A |
| NA | Germany | PD | AF231765 | NA | A |
| NA | USA | 28 | AY752944 | Myocarditis | A |
| NA | USA | 0 | AY752945 | Non-pathogenic | A |
| NA | USA | 20 | AY752946 | Myocarditis | A |
| 2008 | Australia | NSW-V13B-2008 | MF678302 | Tpe 1 diabetes | A |
| 1949 | USA | Nancy | M88483 | Myocarditis | A |
| NA | Germany | PD2 | DQ093616 | NA | A |
| NA | Germany | K28B | DQ093615 | NA | A |
| 1956 | USA | GA | AY673831 | Non-pathogenic | B |
| 1999 | Germany | 99-1000 | DQ093619 | NA | B |
| 1999 | Uzbekistan | CBV3-11059-99 | AY896762 | AFP | C |
| 2009 | India | NIV09001C5LVH7 | JX476161 | AFP asymptomatic contacts | C |
| 2009 | India | NIV09001C1LVH1 | JX476162 | AFP asymptomatic contacts | C |
| 2009 | India | NIV09001C2LVH2 | JX476163 | AFP asymptomatic contacts | C |
| 2009 | India | NIV09001C3LVH4 | JX476164 | AFP asymptomatic contacts | C |
| 2009 | India | NIV09001C4LVH5 | JX476165 | AFP asymptomatic contacts | C |
| 2009 | India | NIV0917801LV243 | JX476167 | AFP | C |
| 2009 | India | NIV099741LV204 | JX476168 | AFP | C |
| 2009 | India | NIV095181LV33 | JX476170 | AFP | C |
| 2009 | India | NIV094331LV26 | JX476171 | AFP | C |
| 2009 | India | NIV099351LV53P4 | KR107054 | AFP | C |
| 2009 | India | NIV0914321LV141P5 | KR107055 | AFP | C |
| 2009 | India | NIV099101LV198P7 | KR107056 | AFP | C |
| 2008 | India | A050D | JX513572 | Diarrhea | C |
| 2009 | India | A210D | JX513573 | Diarrhea | C |
| 2009 | India | A219D | JX513574 | Diarrhea | C |
| 2009 | India | M475 | JX513576 | Diarrhea | C |
| 2009 | India | A79-12a | KF177116 | Diarrhea | C |
| 2011 | Madagascar | MAD9774-11 | KR232787 | NA | C |
| 2011 | Madagascar | MAD9775-11 | KR232788 | NA | C |
| 2015 | China | 2015-082-CVB3 | MW179426 | NA | D |
| 2001 | China | 2001-5 | MK791148 | NA | D |
| 2017 | France | STRAS-17-606-FRA17 | MK561368 | Myopericarditis | D |
| 2017 | France | LYO-VPLA17-37-68-FRA1 | MK561369 | Myocarditis | D |
| 2017 | France | LYO-VLCR17-14-61-FRA17 | MK561370 | Myocarditis and meningo-Encephalitis | D |
| 2017 | France | LYO-VPLA17-29-05-FRA17 | MK561371 | Myocarditis and meningo-Encephalitis | D |
| 2017 | France | LYO-VPLA17-29-07-FRA17 | MK561372 | Myocarditis and meningo-Encephalitis | D |
| 2015 | China | RA/42/15/B3 | MF467347 | AM | D |
| 2015 | China | RA/19/15/B3 | MF467348 | AM | D |
| 2015 | France | CF315253105_FRA15-09-10_CV-B3 | MK086191 | neonate infection | D |
| 2017 | China | XZ17-10/XZ/CHN/2017 | OK643874 | Non-pathogenic | D |
| 2012 | China | GS12-70/GS/CHN/2012 | OK632334 | HFMD | D |
| 2012 | China | GS12-55/GS/CHN/2012 | OK643871 | HFMD | D |
| 2016 | China | GS16-305/GS/CHN/2016 | OK632335 | HFMD | D |
| 2016 | China | GD16-53/GD/CHN/2016 | OK632332 | HFMD | D |
| 2016 | China | GD16-69/GD/CHN/2016 | OK632333 | HFMD | D |
| 2012 | China | HuN12-42/HuN/CHN/2012 | OK632336 | HFMD | D |
| 2015 | China | HuN15-26/HuN/CHN/2015 | OK632337 | HFMD | D |
| 2016 | China | HuN16-54/HuN/CHN/2016 | OK643872 | HFMD | D |
| 2016 | China | XJ16-62/XJ/CHN/2012 | OK632350 | HFMD | D |
| 2016 | China | XJ16-86/XJ/CHN/2016 | OK632351 | HFMD | D |
| 2012 | China | XJ12-36/XJ/CHN/2012 | OK643873 | HFMD | D |
| 2013 | China | SaX13-29/SaX/CHN/2013 | OK632341 | HFMD | D |
| 2013 | China | SaX13-63/SaX/CHN/2013 | OK632342 | HFMD | D |
| 2015 | China | TJ15-78/TJ/CHN/2015 | OK632349 | HFMD | D |
| 2016 | China | JL16-3/JL/CHN/2016 | OK632339 | HFMD | D |
| 2016 | China | JL16-4/JL/CHN/2016 | OK632340 | HFMD | D |
| 2013 | China | JL13-59/JL/CHN/2016 | OK632338 | HFMD | D |
| 2016 | China | SD16-TA150H/SD/CHN/2016 | MH293529 | HFMD | D |
| 2016 | China | SD16-TA164H/SD/CHN/2016 | MH293530 | HFMD | D |
| 2016 | China | SD16-TA178/SD/CHN/2016 | OK632348 | HFMD | D |
| 2016 | China | SD16-BZ161/SD/CHN/2016 | MH293517 | HFMD | D |
| 2016 | China | SD16-DZ148/SD/CHN/2016 | OK632343 | HFMD | D |
| 2016 | China | SD16-DZ155/SD/CHN/2016 | MH293519 | HFMD | D |
| 2016 | China | SD16-HZ276H/SD/CHN/2016 | MH293522 | HFMD | D |
| 2016 | China | SD16-LC100H/SD/CHN/2016 | OK632344 | HFMD | D |
| 2016 | China | SD16-LC114H/SD/CHN/2016 | OK632345 | HFMD | D |
| 2016 | China | SD16-LC115H/SD/CHN/2016 | OK632346 | HFMD | D |
| 2016 | China | SD16-LW041H/SD/CHN/2016 | OK632347 | HFMD | D |
| 2011 | China | XZ2011025 | MH836324 | Non-pathogenic | D |
| 2011 | China | XZ2011028 | MH836323 | Non-pathogenic | D |
| 2009 | China | A103/KM/09 | JX843810 | AM | D |
| 2006 | China | SSM-CVB3 | GU109481 | Myocarditis | D |
| 2008 | China | GZ803 | FJ357838 | NA | D |
| 2012 | China | CVB3SD2012CHN | JX976770 | HFMD | D |
| 2012 | China | PZ23Y/JS/2012 | KP036481 | HFMD | D |
| 2012 | China | Beijing0811 | GQ141875 | Myocarditis | D |
| 2012 | China | DH16G/JS/2012 | KP036480 | HFMD | D |
| 2008 | China | 08TC170 | KR362878 | AM | D |
| 2008 | China | CB3/2035A | KY286529 | NA | D |
| 2008 | China | Fuyang19 | FJ000001 | NA | D |
| 2009 | China | KM06 | KJ020100 | NA | D |
| 2011 | China | AH30 | KC481610 | Encephalitis | D |
| 2012 | China | DH09Y/JS/2012 | KP036479 | NA | D |
| 2010 | [Thailand](https://dict.cn/Thailand) | PMKA0219 | KU574624 | Respiratory infections | D |
| 2013 | China | 143-2H-Sewage-YN-CHN-2013 | AB976076 | Non-pathogenic | D |
| 2008 | China | 012/2008TC/SD/CHN | FJ919564 | Encephalitis | D |
| 2008 | China | 177/2008TC/SD/CHN | FJ919566 | Encephalitis | D |
| 2005 | China | YZ127/SD/CHN/2005/CB3 | GQ246518 | AM | D |
| 2006 | China | AM06HZ/SD/CHN/2006/CB3 | GQ329744 | AM | D |
| 2000 | China | 00190/SD/CHN/2000/CB3 | GQ329745 | AFP | D |
| 2000 | China | 00270/SD/CHN/2000/CB3 | GQ329746 | AFP | D |
| 2000 | China | 00281/SD/CHN/2000/CB3 | GQ329747 | AFP | D |
| 2000 | China | 00284/SD/CHN/2000/CB3 | GQ329748 | AFP | D |
| 2000 | China | 00343/SD/CHN/2000/CB3 | GQ329749 | AFP | D |
| 2000 | China | 00346/SD/CHN/2000/CB3 | GQ329750 | AFP | D |
| 2000 | China | 00359/SD/CHN/2000/CB3 | GQ329751 | AFP | D |
| 2000 | China | 00362/SD/CHN/2000/CB3 | GQ329752 | AFP | D |
| 2001 | China | 01330/SD/CHN/2001/CB3 | GQ329753 | AFP | D |
| 2001 | China | 01338/SD/CHN/2001/CB3 | GQ329754 | AFP | D |
| 2008 | China | H156F/SD/CHN/2008/CB3 | GQ329755 | AFP | D |
| 2008 | China | H019Y/SD/CHN/2008/CB3 | GQ329756 | AFP | D |
| 2002 | China | 02217/SD/CHN/2002/CB3 | GQ329757 | AFP | D |
| 2002 | China | 02225/SD/CHN/2002/CB3 | GQ329758 | AFP | D |
| 2004 | China | 04327/SD/CHN/2004/CB3 | GQ329759 | AFP | D |
| 2004 | China | 04433/SD/CHN/2004/CB3 | GQ329760 | AFP | D |
| 2005 | China | 05280/SD/CHN/2005/CB3 | GQ329761 | AFP | D |
| 2008 | China | 08132/SD/CHN/2008/CB3 | GQ329762 | AFP | D |
| 2008 | China | 08153/SD/CHN/2008/CB3 | GQ329763 | AFP | D |
| 2008 | China | 08197/SD/CHN/2008/CB3 | GQ329764 | AFP | D |
| 2008 | China | 08201/SD/CHN/2008/CB3 | GQ329765 | AFP | D |
| 1994 | China | 94196/SD/CHN/1994/CB3 | GQ329766 | AFP | D |
| 2008 | China | 37010408199/SD/CHN/2008/CB3 | GQ329767 | AFP | D |
| 2008 | China | CB3/SD/sewage/080725 | GU272011 | ES | D |
| 2008 | China | CB3/SD/sewage/080729 | GU272012 | ES | D |
| 2009 | China | CB3/SD/sewage/090528/1-4H | GU272013 | ES | D |
| 2008 | China | 08-2035_verus | JQ042700 | HFMD | D |
| 1996 | China | 96HN+3/SD/CHN/1996 | JQ364844 | AFP | D |
| 2000 | China | 00353/SD/CHN/2000 | JQ364845 | AFP | D |
| 2001 | China | 01281/SD/CHN/2001 | JQ364846 | AFP | D |
| 2001 | China | 01318/SD/CHN/2001 | JQ364847 | AFP | D |
| 2001 | China | 01343/SD/CHN/2001 | JQ364848 | AFP | D |
| 2007 | China | 2007AMES.Cao/SD/CHN | JQ364849 | AM | D |
| 2007 | China | 2007AMES.Yang/SD/CHN | JQ364850 | AM | D |
| 2002 | China | 02153/SD/CHN/2002 | JQ364851 | AFP | D |
| 2002 | China | 02197/SD/CHN/2002 | JQ364852 | AFP | D |
| 2002 | China | 02243/SD/CHN/2002 | JQ364853 | AFP | D |
| 2002 | China | 02251/SD/CHN/2002 | JQ364854 | AFP | D |
| 2002 | China | 02273/SD/CHN/2002 | JQ364855 | AFP | D |
| 2002 | China | 02298/SD/CHN/2002 | JQ364856 | AFP | D |
| 2002 | China | 02302/SD/CHN/2002 | JQ364857 | AFP | D |
| 2003 | China | 03243/SD/CHN/2003 | JQ364858 | AFP | D |
| 2005 | China | 05213/SD/CHN/2005 | JQ364859 | AFP | D |
| 2005 | China | 05267/SD/CHN/2005 | JQ364860 | AFP | D |
| 2005 | China | 05275/SD/CHN/2005 | JQ364861 | AFP | D |
| 2005 | China | 05336/SD/CHN/2005 | JQ364862 | AFP | D |
| 2005 | China | 05367/SD/CHN/2005 | JQ364863 | AFP | D |
| 2005 | China | 05416/SD/CHN/2005 | JQ364864 | AFP | D |
| 2008 | China | 08130/SD/CHN/2008 | JQ364865 | AFP | D |
| 2008 | China | 08131/SD/CHN/2008 | JQ364866 | AFP | D |
| 2008 | China | 08135C4/SD/CHN/2008 | JQ364867 | AFP | D |
| 2008 | China | 08135C5/SD/CHN/2008 | JQ364868 | AFP | D |
| 2008 | China | 08180C2/SD/CHN/2008 | JQ364869 | AFP | D |
| 2008 | China | 08199/SD/CHN/2008 | JQ364870 | AFP | D |
| 2008 | China | 08210/SD/CHN/2008 | JQ364871 | AFP | D |
| 2008 | China | 08211/SD/CHN/2008 | JQ364872 | AFP | D |
| 2008 | China | 08281/SD/CHN/2008 | JQ364873 | AFP | D |
| 2009 | China | 09229/SD/CHN/2009 | JQ364874 | AFP | D |
| 1990 | China | 90052/SD/CHN/1990 | JQ364875 | AFP | D |
| 1993 | China | 93010/SD/CHN/1993 | JQ364876 | AFP | D |
| 1996 | China | 96125/SD/CHN/1996 | JQ364877 | AFP | D |
| 1996 | China | 96169/SD/CHN/1996 | JQ364878 | AFP | D |
| 1996 | China | 96174/SD/CHN/1996 | JQ364879 | AFP | D |
| 1996 | China | 96191/SD/CHN/1996 | JQ364880 | AFP | D |
| 1996 | China | 96226/SD/CHN/1996 | JQ364881 | AFP | D |
| 1996 | China | 96302/SD/CHN/1996 | JQ364882 | AFP | D |
| 2002 | China | 02HZ+4/SD/CHN/2002 | JQ364883 | AFP | D |
| 2002 | China | 02HZ+5/SD/CHN/2002 | JQ364884 | AFP | D |
| 2010 | China | JNEW100429/SD/CHN/2010 | JQ364885 | ES | D |
| 2005 | Taiwan,China | 2005-649 | JQ390179 | NA | D |
| 2005 | Taiwan,China | 2005-812 | JQ390180 | NA | D |
| 2005 | Taiwan,China | 2005-0927 | JQ390181 | NA | D |
| 2005 | Taiwan,China | 2005-960 | JQ390182 | NA | D |
| 2005 | Taiwan,China | 2005-1271 | JQ390183 | NA | D |
| 2005 | Taiwan,China | 2005-1362 | JQ390184 | NA | D |
| 2005 | Taiwan,China | 2005-1396 | JQ390185 | NA | D |
| 2005 | Taiwan,China | 2005-1451 | JQ390186 | NA | D |
| 2005 | Taiwan,China | 2005517 | JQ390189 | NA | D |
| 2001 | Taiwan,China | 20011693 | JQ390190 | NA | D |
| 2003 | Taiwan,China | 20031159 | JQ390191 | NA | D |
| 2004 | Taiwan,China | 20040261 | JQ390192 | NA | D |
| 2004 | Taiwan,China | 20040263 | JQ390193 | NA | D |
| 2004 | Taiwan,China | 20040264 | JQ390194 | NA | D |
| 2004 | Taiwan,China | 20040267 | JQ390195 | NA | D |
| 2004 | Taiwan,China | 20040269 | JQ390196 | NA | D |
| 2004 | Taiwan,China | 20040270 | JQ390197 | NA | D |
| 2004 | Taiwan,China | 20040281 | JQ390198 | NA | D |
| 2005 | Taiwan,China | 20050957 | JQ390199 | NA | D |
| 2005 | Taiwan,China | 20051087 | JQ390200 | NA | D |
| 2000 | Taiwan,China | E2000008 | JQ390202 | NA | D |
| 2005 | Taiwan,China | E2005351 | JQ390203 | NA | D |
| 2005 | Taiwan,China | E2005601 | JQ390204 | NA | D |
| 2005 | Taiwan,China | E2005764 | JQ390205 | NA | D |
| 2005 | Taiwan,China | E2005852 | JQ390206 | NA | D |
| 2005 | Taiwan,China | E2005905 | JQ390207 | NA | D |
| 2005 | Taiwan,China | E20051194 | JQ390208 | NA | D |
| 2005 | Taiwan,China | E20051274 | JQ390209 | NA | D |
| 1999 | Taiwan,China | AFP9911003 | JQ390210 | AFP | D |
| 1999 | Taiwan,China | AFP9911004 | JQ390211 | AFP | D |
| 2007 | Taiwan,China | E2007668 | JQ390212 | NA | D |
| 2008 | Taiwan,China | E20081390 | JQ390214 | NA | D |
| 2008 | Taiwan,China | E20081391 | JQ390215 | NA | D |
| 2008 | Taiwan,China | E20081392 | JQ390216 | NA | D |
| 2008 | Taiwan,China | E20081393 | JQ390217 | NA | D |
| 2008 | Taiwan,China | E20081388 | JQ390218 | NA | D |
| 2010 | Taiwan,China | E2010441 | JQ390219 | NA | D |
| 2008 | China | JB14080141 | KC867083 | HFMD | D |
| 2008 | China | JB14080324 | KC867084 | HFMD | D |
| 2008 | China | JB14080351 | KC867085 | HFMD | D |
| 2008 | China | JB14080176 | KC867086 | HFMD | D |
| 2012 | China | JB141230091 | KC867087 | HFMD | D |
| 2012 | China | JB141230178 | KC867088 | HFMD | D |
| 2012 | China | JB141230182 | KC867089 | HFMD | D |
| 2012 | China | JB141230183 | KC867090 | HFMD | D |
| 2010 | China | JE002/SD/CHN/12/CB3 | KF246751 | AM | D |
| 2011 | China | JN110617.R2-3 | KF747467 | ES | D |
| 2013 | Russia | SPb_5111/13sew/Sar-6/14/RU | KU841462 | NA | D |
| 2013 | Japan | Se4/Fukushima/JPN/2013 | LC012522 | ES | D |
| 2013 | Japan | Se6/Fukushima/JPN/2013 | LC012524 | ES | D |
| 2012 | China | 2012_SJZ12-0289F/HeB/CHN/2012 | KF246647 | HFMD | D |
| 2016 | China | SD2016-DZ171-CVB3_ | MH293520 | HFMD | D |
| 2016 | China | SD2016-HZ202H-CVB3 | MH293521 | HFMD | D |
| 2016 | China | SD2016-JA224-CVB3 | MH293523 | HFMD | D |
| 2016 | China | SD2016-JN325H-CVB3 | MH293524 | HFMD | D |
| 2016 | China | SD2016-WF451-CVB3 | MH293532 | HFMD | D |
| 2016 | China | SD2016-WF598H-CVB3 | MH293533 | HFMD | D |
| 2016 | China | SD2016-WH134-CVB3 | MH293534 | HFMD | D |
| 2012 | China | S002H-SJZ11-0122T | MH293510 | HFMD | D |
| 2012 | China | S143H-SJZKZH12-0032T | MH293511 | HFMD | D |
| 2012 | China | S165R-SJZKZH12-0202F | MH293512 | HFMD | D |
| 2012 | China | S172R-SJZKZH12-0223F | MH293513 | HFMD | D |
| 2012 | China | S176R-SJZKZH12-0232F | MH293514 | HFMD | D |
| 2012 | China | S178R-SJZKZH12-0235F | MH293515 | HFMD | D |
| 2012 | China | S180R-SJZKZH12-0242F | MH293516 | HFMD | D |
| 2012 | China | SJZ12-0573T/HeB/CHN/2012 | KF246648 | HFMD | D |
| 2012 | China | SJZ12-0729F/HeB/CHN/2012 | KF246649 | HFMD | D |
| 2012 | China | SJZ12-0735F/HeB/CHN/2012 | KF246650 | HFMD | D |
| 2012 | China | SJZ12-0772T/HeB/CHN/2012 | KF246651 | HFMD | D |
| 2012 | China | SJZ12-0897F/HeB/CHN/2012 | KF246652 | HFMD | D |
| 2012 | China | SJZ12-0928F/HeB/CHN/2012 | KF246653 | HFMD | D |
| 2012 | China | SJZK12-0012F/HeB/CHN/2012 | KF246654 | HFMD | D |
| 2012 | China | SJZK12-0033F/HeB/CHN/2012 | KF246655 | HFMD | D |
| 2012 | China | SJZK12-0042F/HeB/CHN/2012 | KF246656 | HFMD | D |
| 2012 | China | SJZK12-0045F/HeB/CHN/2012 | KF246657 | HFMD | D |
| 2016 | UK | Env_2016_Sep | MG451802 | ES | E |
| 1993 | France | 2679 | KJ489414 | NA | E |
| 1989 | Romania | RO-69-1-89 | LS451288 | NA | E |
| 1995 | Romania | RO-123-1-95 | LS451287 | NA | E |
| 2002 | Poland | 1500/PL12/2002 | KU189245 | AFP | E |
| 2002 | Poland | 1595/PL20/2002 | KU189246 | AFP | E |
| 2004 | Poland | 1792/PL02/2004 | KU189248 | AFP | E |
| 2008 | Poland | 2048/PL24/2008 | KU189250 | AFP | E |
| 2012 | Russia | SPb_220/12Hel/NAO-18/14/RU | KU841461 | Non-pathogenic | E |
| 2012 | Russia | SPb_219/12Hel/NAO-17/14/RU | KU841460 | Non-pathogenic | E |
| 2013 | Japan | Se5/Fukushima/JPN/2013 | LC012523 | ES | E |
| NA | Germany | 0518/79 | DQ093625 | NA | E |
| NA | Germany | 0141/79 | DQ093633 | NA | E |
| NA | Germany | 0144/81 | DQ093626 | NA | E |
| NA | Germany | 0441/84 | DQ093635 | NA | E |
| NA | Germany | 0113/79 | DQ093632 | NA | E |
| NA | Germany | 0445/81 V | DQ093634 | NA | E |
| NA | Germany | 0142/78 | DQ093631 | NA | E |
| NA | Germany | 0450/76 | DQ093624 | NA | E |
| NA | Germany | 0412/87 | DQ093627. | NA | E |
| NA | Germany | 0513/87 | DQ093636 | NA | E |
| NA | Germany | 1569/70 | DQ093630. | NA | E |
| NA | Germany | 1340/70 | DQ093629 | NA | E |
| 2000 | Germany | 00-637 | DQ093618 | NA | E |
| 1997 | Germany | 97-927_1207K | JX946654 | NA | E |
| 1997 | Germany | 97-927_1207R | JX946655 | NA | E |
| 1997 | Germany | 97-927 | DQ093617 | NA | E |
| 1998 | Germany | 98-1422 | DQ093628 | NA | E |
| NA | Germany | 304037 | DQ093620 | NA | E |
| NA | Germany | 390557 | DQ093623 | NA | E |
| NA | Germany | 302784 | DQ093621 | NA | E |
| NA | Germany | 392323 | DQ093622 | NA | E |
| 2005 | USA | CVB3-MCH | EU144042 | Myocarditis | E |
| 2019 | USA | 2019-23309 | MN896912 | NA | E |
| 2015 | France | CF315241021_FRA15-08-28_CV-B3 | MK086188 | ES, fever | E |
| 2006 | France | CVB3_CF209062_FRA06 | HF948088 | NA | E |
| 2008 | Taiwan,China | E20081389 | JQ390213 | NA | E |
| 2000 | Taiwan,China | AFP2000250 | JQ390201 | AFP | E |
| 2018 | USA | 23091 | MK652138 | NA | E |
| 2009 | Australia | NSW-V22-2009 | MF678311 | Tpe 1 diabetes | E |
| 2015 | Australia | NSW-V25-2015 | MF678314 | Tpe 1 diabetes | E |
| 2006 | Australia | NSW-V35B-2006 | MF678324 | Tpe 1 diabetes | E |
| 2013 | Australia | NSW-V37-2013 | MF678326 | Tpe 1 diabetes | E |
| 2009 | Australia | NSW-V38A-2009 | MF678327 | Tpe 1 diabetes | E |
| 2009 | Australia | NSW-V39-2009 | MF678328 | Tpe 1 diabetes | E |
| 2006 | Australia | NSW-V40-2006 | MF678329 | Tpe 1 diabetes | E |
| 2012 | Australia | NSW-V15-2012 | KC867048 | Tpe 1 diabetes | E |
| 2008 | Indonesia | 2008910445 | KR054737 | NA | E |
| 2010 | [Thailand](https://dict.cn/Thailand) | PMKA0906 | KU574623 | Rspiratory infections | E |
| 2005 | France | CF1851041-05 | AM236933 | AM | E |
| 2005 | France | CF1880311-05 | AM236936 | Myopericarditis | E |
| 2005 | France | CF1930611-05 | AM236938 | AM | E |
| 2005 | France | CF1990111-05 | AM236943 | Sudden infant death syndrom | E |
| 2005 | France | CF2031011-05 | AM236944 | AM | E |
| 2005 | France | 203029-05 | AM711004 | AM | E |
| 2005 | France | 298098-05 | AM711026 | AM | E |
| 2005 | Australia | 05.188.2226 | FJ868286 | NA | E |
| 2006 | Australia | 05.350.3337 | FJ868287 | NA | E |
| 2006 | Australia | 06.006.3430 | FJ868327 | NA | E |
| 2006 | Australia | 06.007.1075 | FJ868328 | NA | E |
| 2006 | Australia | 06.073.4410 | FJ868329 | NA | E |
| 2000 | Taiwan,China | CL2000139 | JQ390174 | NA | E |
| 2000 | Taiwan,China | CL2000-140 | JQ390175 | NA | E |
| 2000 | Taiwan,China | CL2000141 | JQ390176 | NA | E |
| 2000 | Taiwan,China | CL2000-142 | JQ390177 | NA | E |
| 2000 | Taiwan,China | CL2000143 | JQ390178 | NA | E |
| 2000 | Taiwan,China | 2000758 | JQ390187 | NA | E |
| 2000 | Taiwan,China | 2000802 | JQ390188 | NA | E |
| 2001 | France | CVB3_CF3109_FRA01 | HF948084 | NA | E |
| 2014 | France | CF314281026_FRA14-10-06_CV-B3 | MK086263 | AM | E |
| 2010 | Denmark | Pan_troglodytes | JN979570 | Myocarditis | E |
| 2004 | France | CVB3_CF295067_FRA04 | HF948085 | NA | E |
| 2002 | Moldova | CBV3-18219-02 | AY896763 | meningitis | E |
| 2020 | China | GD-2020-EV-274 | OK632352 | HFMD | E |
| 2020 | China | GD-2020-EV-323 | OK643877 | HFMD | E |
| 2020 | China | GD-2020-EV-443 | OK643878 | HFMD | E |
| 2020 | China | GD-2020-EV-596 | OK643879 | HFMD | E |
| 2020 | China | GD-2020-EV-669 | OK643880 | HFMD | E |
| 2020 | China | GD-2020-EV-712 | OK632353 | HFMD | E |
| 2020 | China | GD-2020-EV-716 | OK643881 | HFMD | E |
| 2020 | China | GD-2020-EV-782 | OK643882 | HFMD | E |

NA: no data available

# ES: [environmental](https://dict.cn/environmental%20test) surveillance

# AFP: acute flaccid paralysis

# AM: aseptic Meningitis

# HFMD: hand, foot, and mouth disease
