# Supplementary figures and images for: Whole-genome analysis of coxsackievirus B3 reflects its genetic diversity in China and worldwide
Source: Virol J. 2022 Apr 18;19:69. doi: 10.1186/s12985-022-01796-0 (PMC9014606; doi:10.1186/s12985-022-01796-0)

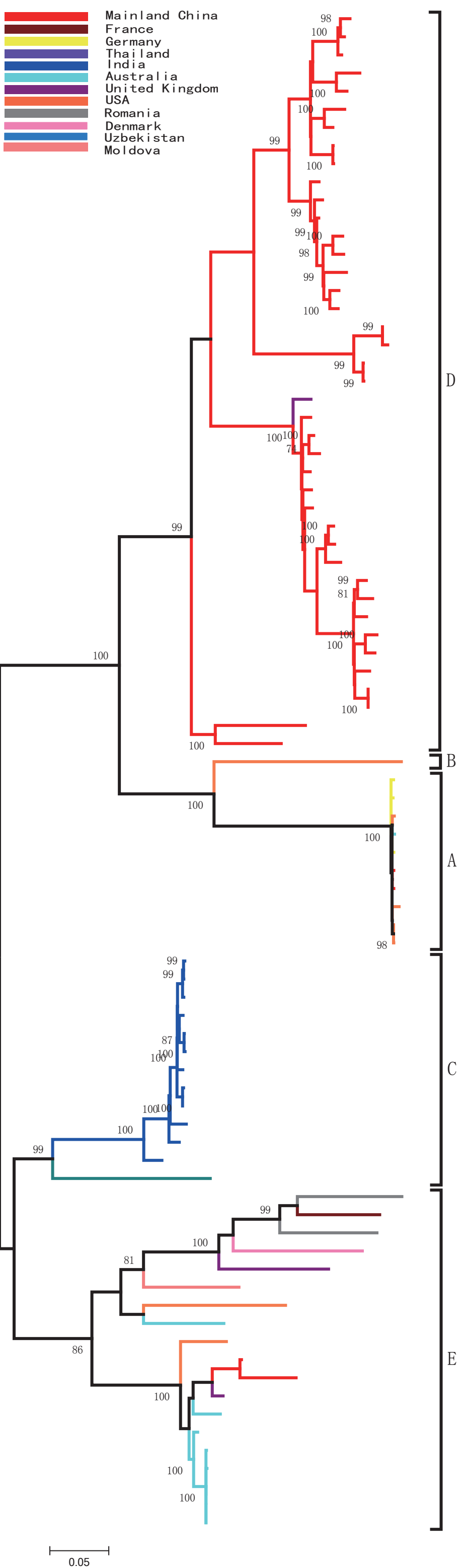

Supplement: Supplementary file 2 — Additional file 2: Fig. S1. A flow chart to show CVB3 sequences used in this analysis. [file 12985_2022_1796_MOESM2_ESM.pdf]

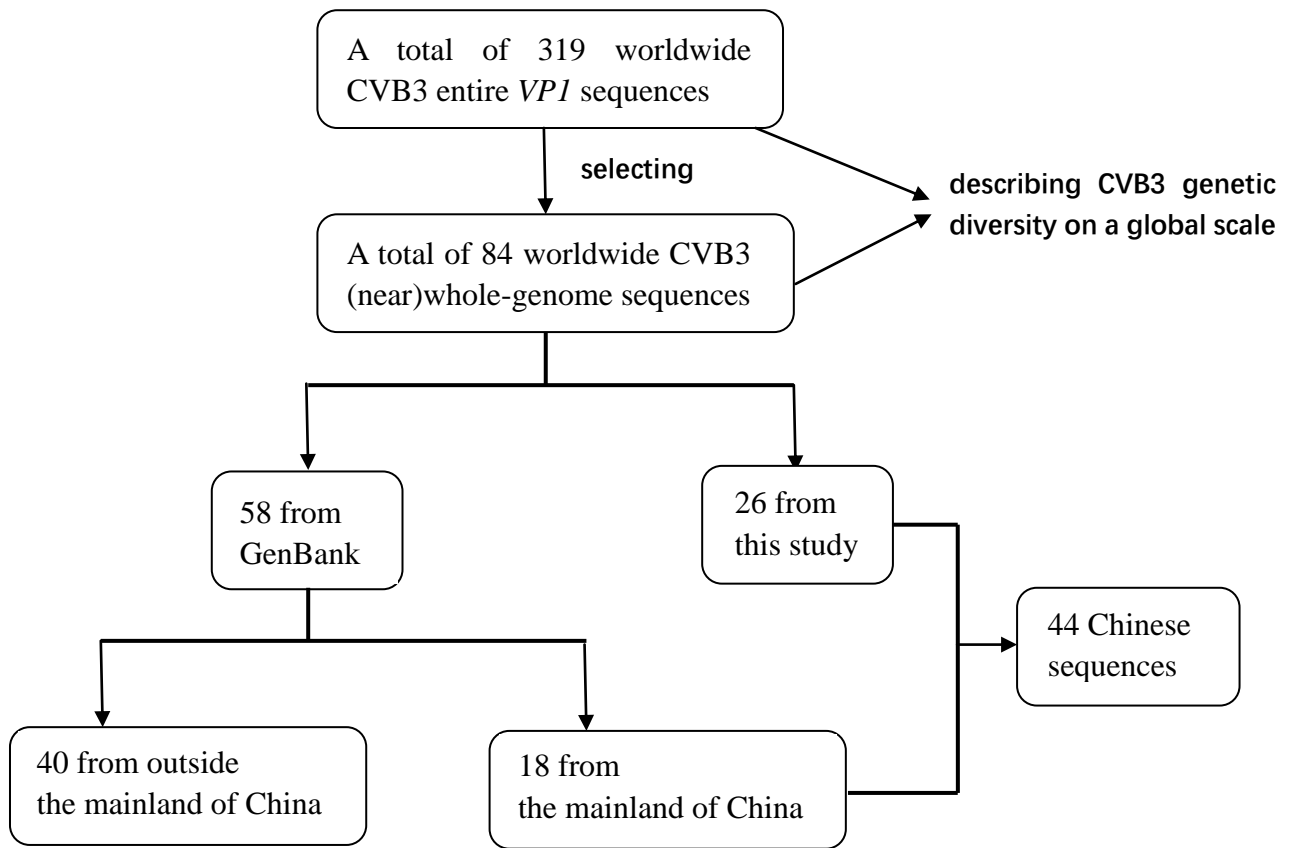

Supplement: Supplementary file 3 — Additional file 3: Fig. S2. The maximum likelihood phylogenetic tree based on the ORF1 sequences (close to the whole genome) of 84 CVB3 genome sequences. [file 12985_2022_1796_MOESM3_ESM.pdf]
